# Treatment and practical considerations of IgA nephropathy

**DOI:** 10.3389/fmed.2026.1911077

**Published:** 2026-10-14

**Authors:** Hanny Sawaf, Junglee Kim, Ali Mehdi, Corey Cavanaugh, Jonathan J. Taliercio

**Affiliations:** Cleveland Clinic Foundation, Department of Kidney Medicine, Cleveland, OH, United States

**Keywords:** IgA nephropathy, a proliferation inducing ligand (APRIL), b-cell activation factor (BAFF), complement inhibition, endothelin receptor antagonist, SGLT2 (sodium-glucose cotransporter 2) inhibitor, RAS blockade

## Abstract

IgA nephropathy is a common cause of glomerular disease that may result in kidney failure. Until recently, angiotensin blockade using angiotensin converting enzyme (ACE) inhibitors and angiotensin receptor blockers (ARB) were the main treatment options for patients with IgA nephropathy. Newer drugs such as sodium-glucose co-transporter 2 (SGLT2) inhibitors, endothelin angiotensin receptor antagonists (DEARA), endothelin receptor antagonists (ERA), complement inhibitors, targeted release formulation glucocorticoids, and a proliferation-inducing ligand (APRIL) and B cell activation factor (BAFF) inhibitors have shown promise in reducing proteinuria and delaying the progression of IgA nephropathy. In this review, we will explore the latest pharmacological options, their efficacy, and when they should be considered in adult patients with IgA nephropathy.

## 1 Introduction

IgA nephropathy is the most common cause of primary glomerulopathy worldwide with an estimated incidence of 2.5 cases per 100,000 persons (1). It typically occurs in White and East Asian patients 20–40 years of age and has a risk for disease progression to kidney failure within 10–15 years of diagnosis (2–5).

Regardless of the trigger (infection, exogenous mucosal antigens, gut microbiome) the pathophysiology of IgA nephropathy is characterized by a four “Hit” immunological process which starts with increased production of galactose deficient IgA1 (Gd-IgA) (Hit 1) then production of IgA and/or IgG autoantibodies against Gd-IgA (Hit 2) (6, 7). These Gd-IgA and their autoantibodies form pathogenic immune complexes (Hit 3). Mesangial deposition of these immune complexes results in inflammation, complement activation, and glomerular injury (Hit 4) (6–8).

New FDA approved drugs have given healthcare providers and patients several treatment options which are categorized into non-immunosuppressive and immunosuppressive therapies, which can be used alone or in combination. Depending on the medication used, these therapies can be utilized as either disease specific or nephron-sparing therapies. Non-immunosuppressive drugs include angiotensin converting enzyme (ACE) inhibitors and angiotensin receptor blockers (ARB), sodium-glucose transport protein 2 (SGLT2) inhibitors, endothelin receptor antagonists (ERA), and dual endothelin angiotensin receptor antagonists (DEARA) (9–12). Immunosuppressive drugs that target the “Hit 1” pathway include targeted release budesonide, and a proliferation-inducing ligand (APRIL) and B cell activation factor (BAFF) inhibitors such as sibeprenlimab and atacicept (13, 14). Iptacopan, a complement Factor B inhibitor, targets “Hit 4” by reducing complement activation in the circulation and mesangium caused by the deposition of preformed immune complexes (15).

This review focuses on understanding the mechanism of these therapeutic options, when they should be considered, and other considerations when prescribing these agents in adults (Figure 1).

**Figure 1:**
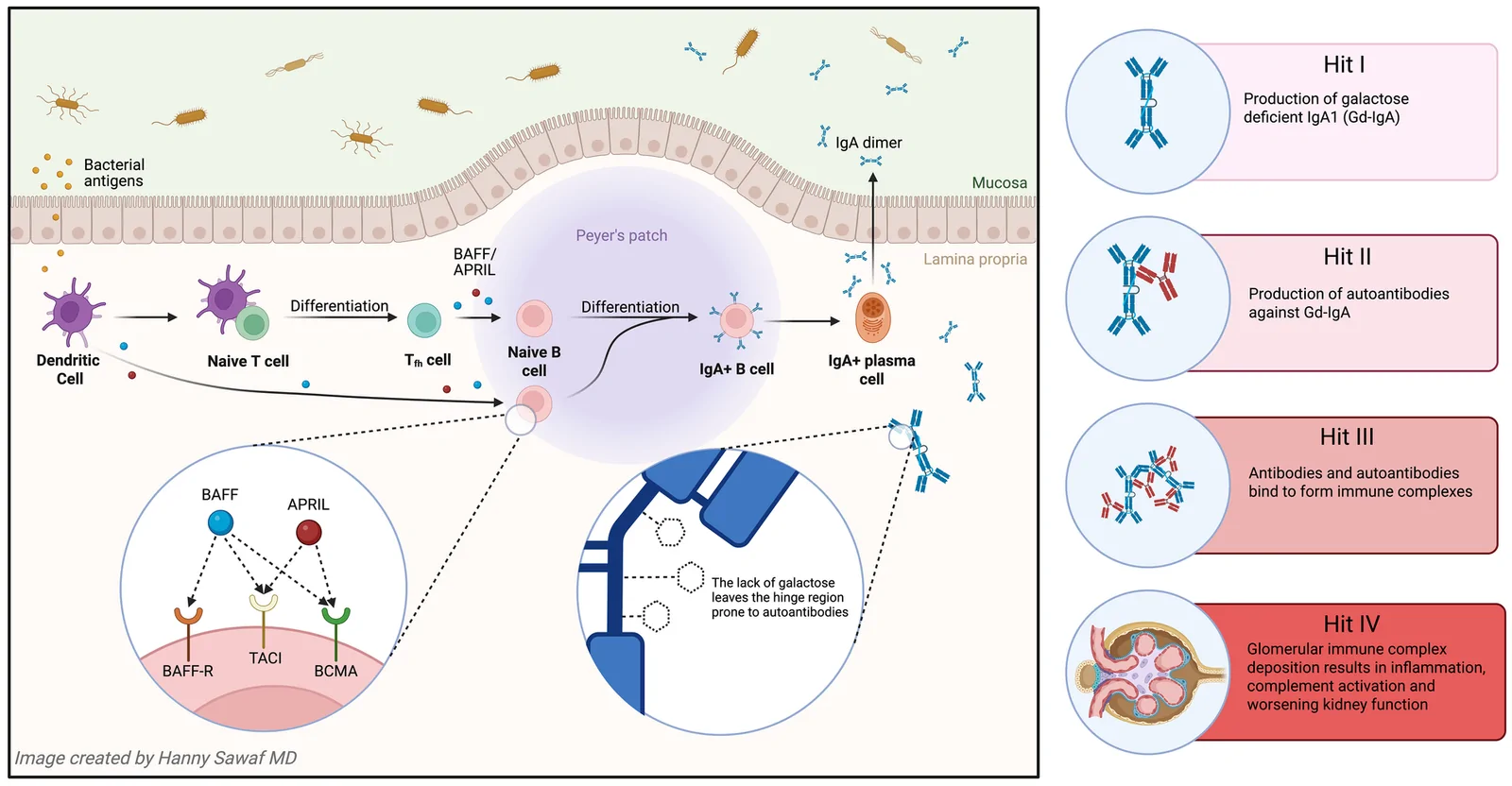
Visual representation of the pathophysiology of IgA nephropathy and the 4-Hit hypothesis.

## 2 ACE inhibitors and ARBs

### 2.1 Introduction

Multiple studies have demonstrated that ACE inhibitors and ARBs reduce CKD progression in patients with IgAN, especially in the presence of proteinuria > 0.5 g/g (10, 13, 14). The upregulation of renin angiotensin system (RAS) particularly through the vasoactive angiotensin 2 has pro-fibrotic and pro-inflammatory effects (16–18). ACE inhibitors and ARBs have been shown to provide renal protection through renal efferent arteriolar vasodilation, decreasing glomerular hyperfiltration and hypertension, as well as other mechanisms (16, 19).

### 2.2 Practical considerations

RAS inhibition is the cornerstone of all proteinuric chronic kidney disease. Kidney protective evidence of RAS blockade in non-proteinuric CKD is less robust. However, international societies recommend that all patients (regardless of proteinuria) receive maximally tolerated ACE inhibitor or ARB. They also cautiously recommend using ACE/ARB in normotensive patients. As is done in other forms of proteinuric kidney diseases, it is generally agreed to utilize these agents and increase the dose of ACE inhibitors or ARBs to the maximal tolerated dose (20–22).

Initiation of ACE or ARB may cause a decline in eGFR of up to ∼30% due to decreasing intraglomerular pressure. Therefore, re-assessing kidney function at initiation of RAS blockade and during dose titration should occur within 2–4 weeks of change. Cessation of RAS blockade should reverse the rise in serum creatinine. Declines in eGFR > 30% may not require cessation of therapy, but should warrant further investigation to assess volume depletion, concomitant medications such as diuretics and NSAIDS, or renal artery stenosis. Checking a metabolic panel 2–4 weeks after initiation of an ACE inhibitor or ARB can be helpful to monitor the kidney function (23).

Hyperkalemia is a common electrolyte disorder when using RAS blockade, especially in patients with advanced kidney disease. Persistent hyperkalemia of ≥6 mEq/L not amendable to medical therapy should warrant permanently stopping RAS blockade therapy. Mitigating the hyperkalemia risk while allowing maximal RAS blockade would include ensuring patients are not taking potassium supplements or NSAIDS (23, 24). Dietary potassium review to ensure processed food restriction, avoiding constipation, treating metabolic acidosis and hyperglycemia, using a diuretic for kaliuresis, or utilizing an approved potassium binding agent such as patiromer can also be considered (25, 26). SGLT2 inhibitors, discussed below, have been shown to decrease the risk of hyperkalemia and to allow continued RAS blockade (27–29).

Cough (3.9%–35%) and angioedema (0.1%–0.7%) are more commonly seen in ACE inhibitors due increased bradykinin levels and may occur at any time during treatment. This side effect is much less common in ARBs although it has been described in rare instances (30–32). AHA/ACC guidelines recommend switching to an ARB for ACE inhibitor provoked cough or angioedema (26). If a patient fails ARB challenge, or through a shared decision decides not to re-challenge, another consideration could be a mineralocorticoid receptor antagonist as they have shown benefit in nondiabetic CKD and glomerular disease (33–37).

ACE inhibitors and ARBs are contraindicated in pregnancy due to their teratogenic effects, therefore shared decision making regarding effective contraception methods should be discussed. If RAS blockade is clinically required during breast feeding, enalapril has shown to be safe (38, 39) (Table 1).

**Table 1:** Practical considerations for RAS inhibitors.

Medication	Practical consideration
RAS Inhibitors	**Hyperkalemia:** Monitor with lab draws. Some considerations include low potassium diet, treatment of constipation, potassium binders and utility of SGLT2 inhibitors **Decline in eGFR:** Monitor with lab draws. **Teratogenicity:** Rule out pregnancy and consider contraception before drug initiation. **Angioedema and cough with ACE inhibition:** Stop medication and consider switching to ARB **Combination therapy:** Do not combine ACE inhibitors with ARB therapy.

### 2.3 Evidence and utility

Proteinuria is an important risk factor for patients with IgA nephropathy where higher levels of proteinuria are associated with higher rates of disease progression (40). Small studies showed potential benefits of RAS inhibition in IgA nephropathy (41, 42). Meta-analysis looking at such trials also revealed similar beneficial findings (43, 44). The Hong Kong study using valsartan in IgA Nephropathy (HKVIN) conducted a randomized placebo controlled trial comparing valsartan to placebo in patients with IgA nephropathy showing that valsartan decreased proteinuria and slowed the rate of progression of patients with IgA nephropathy (10).

Even with the current advancements and new agents for IgA nephropathy, RAS inhibition remains first line and all patients should be on maximum tolerated or labeled ACE inhibition or ARB (45).

## 3 SGLT2 inhibitors

### 3.1 Introduction

SGLT2 inhibitors are oral medications initially approved to lower blood sugar in adults with type 2 diabetes by enhancing glucosuria (46). SGLT2 inhibitors lower hemoglobin A1C by on average of 0.5%–1%, and result in weight loss of 1–3 kg. This class of medications have cardiovascular and kidney benefits, in patients with and without diabetes (47, 48).

SGLT2 inhibitors provide renal protection through the tubuloglomerular feedback mechanism. Inhibition of the SGLT2 receptor leads to glucosuria and natriuresis which results in distal sodium delivery. The presence of increased sodium and chloride concentration at the macula densa in the distal convoluted tubule leads to vasoconstriction of the afferent arteriole and decreased intraglomerular hypertension resulting in renal protection (49, 50). Additional renal protection mechanisms include decreasing renal parenchymal oxidative stress, fibrosis and inflammation (51, 52). SGLT2 inhibitors also induce a mild state of ketosis which is also thought to provide renal and cardiac protection (53).

### 3.2 Practical considerations

SGLT2 inhibitors have been shown to be safe and effective in eGFR ≥ 20 mL/min/1.73m^2^ and patients should remain on them until dialysis initiation (47, 54, 55).

Initiation of SGLT2 inhibitors often results in an acute drop in eGFR due to the protective afferent arterial vasoconstriction leading to decrease intraglomerular hypertension. The acute declines in eGFR may be more disconcerting when SGLT2 inhibitors are initiated in more advanced kidney disease and therefore preemptive patient counseling is important. Studies consistently demonstrate that the eGFR benefits occur after 1 year of therapy (56). Checking a metabolic panel 2–4 weeks after medication initiation can be considered for this. Increasing ACE inhibitors or ARBs to the maximally tolerated or labeled dose prior to initiation of an SGLT2 inhibitor should be prioritized (47, 48, 56).

Studies have demonstrated that there is a negligible risk of hypoglycemia in nondiabetics patients, but that risk may be increased in patients with diabetes who are on concomitant medications such as sulfonylurea and/or insulin (57, 58). Therefore, in patients with well controlled diabetes, dose adjustment of insulin or an insulin secretagogue and close monitoring of glucose levels may be required upon initiation of an SGLT2 inhibitor.

Due to their natriuretic effect, SGLT2 inhibitors are effectively weak diuretics. As such, it is advisable to review the patient's diuretic regimen and consider adjustments at the time of initiation. A “sick day protocol” can also be considered in the setting of acute illness where the risk of volume depletion is high such as with diarrhea, vomiting or poor oral intake (54). The risk of diabetic ketoacidosis (DKA) is also a concern in these situations.

SGLT2 inhibitors have also been linked to an increased risk of infections including mycotic infections, urinary tract infections (UTI) and in rare instances Fournier's gangrene. This is likely related to the increased glucose concentration in the lower urinary tract. In addition to patient education on these symptoms, the risk can be reduced with self-care practices of daily hygiene in addition to rinsing the genital area with water after every void and before going to bed (53).

It has been hypothesized that some of the cardiac and renal benefits of SGLT2 inhibitors are a result of a metabolic shift that results in low grade, or therapeutic ketonemia. Although rare and pathologic euglycemic DKA has been reported in up to 2.6% of patients who take SGLT2 inhibitors (59). Patients may present with metabolic acidosis, ketonemia, and near normal blood glucose <250 mg/dL and treatment would require stopping the SGLT2, intravenous fluids, and insulin. For this reason, caution is advised in patients with a history of DKA. Furthermore, this is a major reason these medications have not been approved in the United States for patients with type 1 diabetes (60). Finally, there was concern of increased risk of lower limb amputations due to findings in the CANVAS trial however these findings were not seen in follow up clinical trials (47, 48, 56, 61) (Table 2).

**Table 2:** Practical considerations for SGLT2 inhibitors.

Medication	Practical consideration
SGLT2 Inhibitors	**Expected decline of eGFR upon initiation:** Monitor with lab draws. **Mycotic infections:** Proper postcoital and bathroom hygiene. **DKA:** Not approved in patients with Type 1 diabetes. Caution or avoidance in patients with a history of DKA. **Hypoglycemia:** Monitor concomitant insulin and/or insulin secretagogues dose. **Hypovolemia:** Monitor concomitant diuretic dose. Implement “sick day protocol” in the setting of acute illness causing poor oral intake, nausea, vomiting and/or diarrhea.

### 3.3 Evidence and utility

SGLT2 inhibitors were initially studied for patients with diabetes (56). Follow up land mark trials investigated a broader range of patients including patients with CKD without a diagnosis of diabetes (47, 48). Follow up sub-analysis was done amongst patients from DAPA-CKD and EMPA-Kidney specifically looking into those with IgA nephropathy and the benefits of SGLT2 inhibition held in that patient population (11, 62). These findings led to SGLT2 inhibitors being included in the newest KDIGO guidelines for IgA nephropathy (25).

Given their proven effectiveness in delaying the progression of CKD, SGLT2 inhibitors should be considered in patients with proteinuria despite being on maximum tolerated or labeled ACE inhibitor or ARB therapy. KDIGO recommendations do point out that there is some uncertainty when it comes to younger patients with a preserved renal function and those without proteinuria (45). There may be additional benefit when used in patients with overweight or obesity (63).

## 4 Endothelin receptor antagonists

### 4.1 Introduction

Renal endothelial cells are the major source of endothelin, a potent vasoconstrictor, which has been shown to disrupt podocyte cytoskeleton and promote apoptosis and fibrosis. Its production is increased in various conditions such as diabetes, obesity, FSGS, and IgA nephropathy and contributes to CKD progression (64–66). Renal endothelin-1 preferentially contracts the efferent arteriole via agonisms of endothelin receptor A (ETA). ETA receptor antagonists have been shown to reduce blood pressure and promote renal vasodilatation contributing to protective downstream effects (67–69). Endothelin and angiotensin have also been found to exacerbate each other's actions (70–72).

Atrasentan is a highly selective endothelin receptor antagonist while sparsentan provides dual antagonism by blocking both ETA and the angiotensin II receptor AT1 (73, 74). These two novel agents have been shown to reduce proteinuria when compared to RAS inhibition alone, and to date sparsentan has also been shown to reduce the progression of kidney disease (Figure 2).

**Figure 2:**
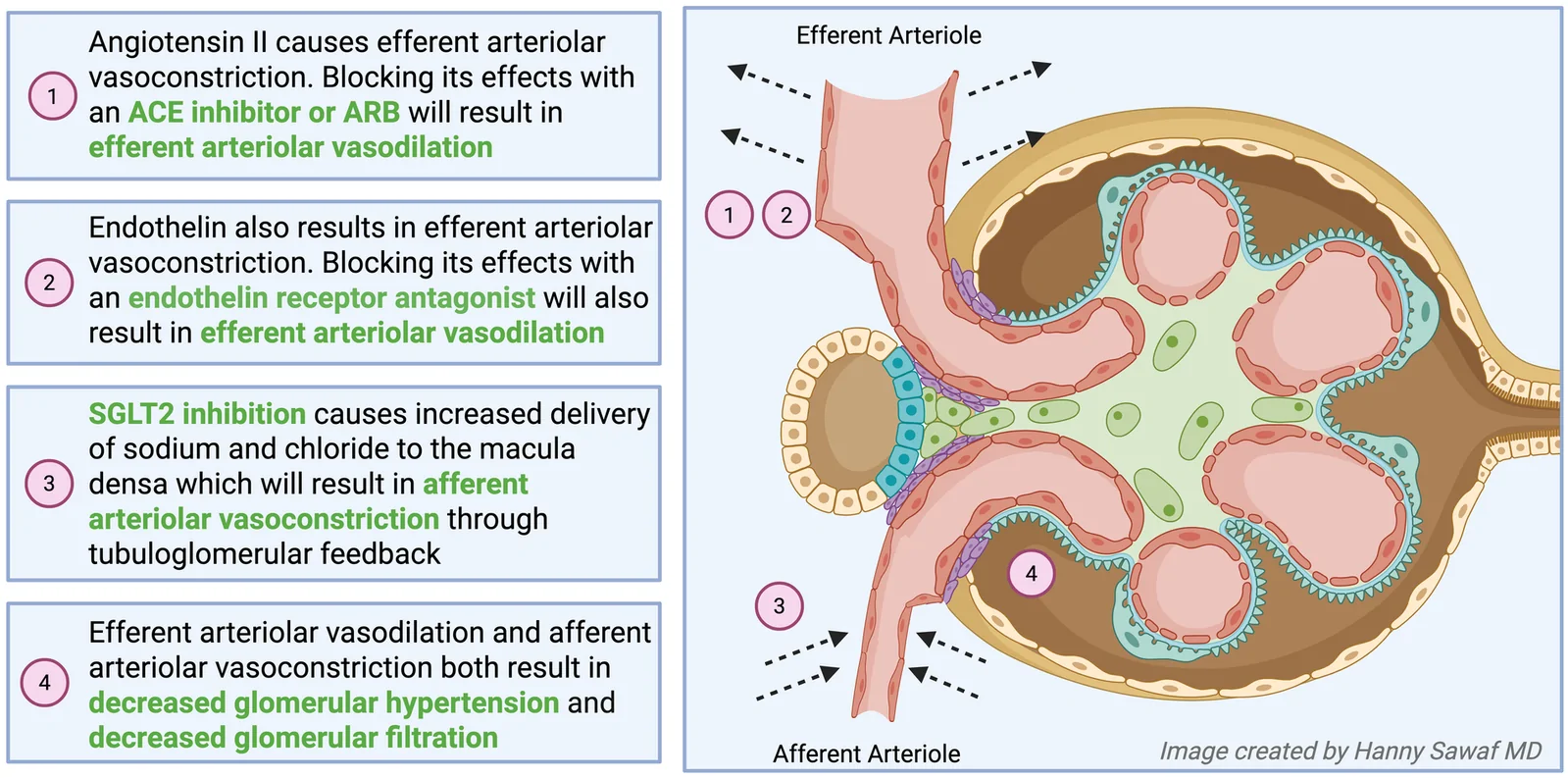
Visual representation on the effect of an angiotensin-converting enzyme (ACE) inhibitors, angiotensin receptor blockers (ARB), sodium-glucose co-transporter 2 (SGLT2) inhibitors, and endothelin receptor antagonists (ERA).

### 4.2 Practical considerations

Upon initiation of ERAs, patients may experience swelling attributed to the increased vascular permeability through the activation of endothelin receptor B (75). For this reason, common causes of volume overload such as nephrotic syndrome and heart failure were excluded from landmark ERA trials (76, 77). Healthcare providers should counsel patients about the possibility of volume overload and may consider adding diuretics to reduce the risk.

Early-era ERA had an increased risk of hepatotoxicity due to the drug's sulfonamide structure. Therefore, sparsentan requires enrollment in Risk Evaluation and Mitigation Strategies (REMS) program to enforce the need for liver function assessment (ALT, AST, bilirubin) at baseline and every 3 months despite the PROTECT trial not showing a significant increased risk of hepatotoxicity (12). Atrasentan does not have a sulfonamide structure and therefore even though liver monitoring is still recommended this does not need to be done through a REMS program.

ERAs have also been found to have teratogenic effects. For this reason, pregnancy should be ruled out in females of child bearing age when starting an ERA (78). As is recommended when initiating RAS inhibitors, contraceptive use should be encouraged in women of childbearing age when starting an ERA.

Anemia has also been described in patients receiving ERAs (79). This adverse effect could be related to changes in iron trafficking (80). Checking a complete blood count (CBC) prior to initiation of an ERA can be helpful. Some trials excluded patients with a hemoglobin less than 9 g/dL or recent blood transfusion requirement within the last 3 months for anemia (76). Checking hemoglobin 3 months after treatment or if clinically indicated can also be considered (81).

Even though atrasentan and sparsentan both result in endothelin receptor antagonism, they are mechanistically different from one another in that sparsentan also blocks AT1. This difference in their mechanism of action also results in some differences that need to be kept in mind when prescribing one of these medications (73, 74). Since sparsentan also blocks AT1, ACE inhibitors and ARBs should be discontinued prior to its initiation. Furthermore, as is seen with ACE inhibitors and ARBs, monitoring potassium levels for signs of hyperkalemia and monitoring eGFR for a greater-than-expected change should be considered (see section on ACE inhibitors and ARBs).

Although ERAs have shown effects on spermatogenesis (82) this side effect has not been demonstrated in sparsentan (83). The FDA label for atrasentan includes a warning for decreased sperm count while that side effect is not included in the FDA label for sparsentan. Therefore, the adverse effect of decreased sperm counts should be considered when prescribing atrasentan (Table 3).

**Table 3:** Practical consideration for endothelin receptor antagonists.

Medication	Practical consideration
All Endothelin Receptor Antagonists	**Volume overload:** Use cautiously in patients at risk of volume overload. **Liver injury:** Monitor liver enzymes prior to initiation; then every 3 months while receiving therapy. **Anemia:** Check hemoglobin prior to initiation and at 3 months or as clinically indicated. **Teratogenicity:** Contraindicated in pregnancy. Consider contraception before drug initiation.
Sparsentan	**Angiotensin II blockade:** Do not combine with ACE inhibitor or ARB. Monitor potassium and creatinine levels 2–4 weeks after initiation. **REMS:** Required for prescription in the United States for hepatic function monitoring.
Atrasentan	**Fertility:** Discuss possible decreased sperm count with male patients.

### 4.3 Evidence and utility

In the PROTECT trial, sparsentan was compared to maximally tolerated irbesartan in patients with biopsy-proven primary IgA nephropathy with an eGFR ≥ 30 mL/min/1.73 m^2^ and a urine protein excretion value of ≥1 g/d despite stable ACE inhibitor or ARB therapy. At 110 weeks, patients receiving sparsentan had an eGFR slope of −2.9 mL/min/1.73 m^2^ per year while those receiving irbesartan had a slope of −3.9 mL/min/1.73 m^2^. The urine protein to creatinine ratio of the sparsentan arm experienced a change of −42.8% compared to −4.4% for the irbesartan arm even though the maximal labeled dose of irbesartan was reached in an impressive 97% of patients (12).

Atrasentan was compared to placebo in the ALIGN trial. Patients had biopsy proven IgA nephropathy with an eGFR ≥ 30 mL/min/1.73 m^2^ and a urine protein excretion value of ≥1 g/d despite stable ACE inhibitor or ARB therapy for at least 12 weeks prior to screening. At week 36, the urine protein to creatinine ratio of the atrasentan arm experienced a change of −38.1% compared to −3.1% for the placebo arm with more recent data at 136 weeks, 4 weeks after the end of the study treatment, showing a difference of 2.39 mL/min/1.73 m^2^ in estimated change in eGFR from baseline vs. placebo (9, 84).

Even though RAS should be prioritized, there remains a role for ERAs in patients with IgA nephropathy. SGLT2 inhibitor and ERA combination is being evaluated for safety and efficacy in the PROTECT open-label extension and the SPARTACUS trial. ERAs should be considered in patients with an eGFR ≥ 30 mL/min/1.73 m^2^ and a urine protein excretion value of ≥1 g/d despite maximal RAS inhibition especially when immunosuppression is not preferred (45).

## 5 Systemic glucocorticoids and targeted steroid formulations

### 5.1 Introduction

Until the advent of new therapies, systemic glucocorticoids had been the mainstay immunosuppressive option for IgA nephropathy however they came with deleterious side effects (85). Systemic steroids broadly act upon all parts of the 4 HIT IgA nephropathy model with evidence showing their ability to reduce Gd-IgA levels, decrease proteinuria and improve renal outcomes (86–88).

Targeted-release budesonide (TRF-budesonide) is an oral steroid that reduces Gd-IgA levels. Theoretically, oral TRF-budesonide is designed to work directly on the Peyer's patches in the distal ileum, a major site of Gd-IgA1 antibodies production (89). The FDA approval of TRF-budesonide has rejuvenated interest in corticosteroid therapy in IgA nephropathy (45).

### 5.2 Practical considerations

Due to the side effect profile of systemic glucocorticoids, assessing patient specific risk factors is critical prior to initiation. TRF-budesonide may be preferred compared to systemic glucocorticoid in appropriate patients, given the reduced systemic exposure although absorption can be variable and studies have shown that steroid-related toxicities should still be anticipated and monitored (90).

TRF-budesonide has received FDA approval for use in patients with IgA nephropathy at risk of disease progression (90). When TRF-budesonide is unavailable or not tolerated, systemic glucocorticoids can be considered for a total treatment duration of 6–9 months (85, 87).

Patients should be counseled on common manifestations of steroid-related hypercortisolism including hypertension, peripheral edema and hyperglycemia. Blood pressure and blood glucose should be closely monitored for this reason. In addition, hypothalamic-pituitary-adrenal axis suppression could happen with prolonged exposure. A gradual taper and in select patients assessment of morning cortisol can help guide the discontinuation of this medication and monitoring for signs of adrenal insufficiency (91).

The effect of glucocorticoids on bone health has also been well established. Assessing baseline fracture risk prior to initiation is important as bone loss is most rapid within the first 6–12 months of therapy. Calcium and vitamin D levels should be assessed and supplemented if needed and bone mineral density testing should be considered in the appropriate setting (92).

As immunosuppressive agents, glucocorticoids increase the susceptibility to infection and may exacerbate underlying latent infections (93). Pneumocystis jirovecii pneumonia (PJP) prophylaxis should be considered in patients receiving prolonged moderate to high dose systemic glucocorticoids (dose equivalent to ≥20 mg prednisone daily over at least a month) with trimethoprim-sulfamethoxazole as the preferred agent (1 DS daily or 1 SS daily). Alternatives include dapsone, atovaquone or inhaled pentamidine (93, 94).

Additional considerations include gastrointestinal toxicity as glucocorticoids may worsen peptic ulcer disease especially when combined with NSAIDS (95). Glucocorticoids may also exacerbate psychiatric symptoms including mood changes, anxiety, depression and steroid-induced psychosis warranting caution in patients with underlying psychiatric disease (96). With respect to pregnancy, first trimester exposure may be associated with an increased risk of cleft lip and palate although evidence is limited. TRF-budesonide has not been studied in pregnant patients so its use is generally not advised (97).

TRF-budesonide is primarily metabolized through CYP3A4 so drug-drug interactions can occur with medications that inhibit or induce CYP3A4 (98). Furthermore, given extensive hepatic metabolism, targeted-release budesonide is generally avoided in patients with advanced liver disease. Given that TRF-budesonide acts the Payers patches, this capsule should be taken whole at least 1 h before breakfast. This capsule should be swallowed whole and not crushed, chewed or opened (Table 4).

**Table 4:** Practical considerations for glucocorticoids.

Medication	Practical consideration
All Glucocorticoids	**Latent infections:** Rule out viral hepatitis or TB prior to initiation. **Adrenal insufficiency:** Tapering of medications prior to discontinuation. **Patients at increased risk of toxicity:** This includes diabetes, pre-diabetes, obesity, active peptic ulceration, uncontrolled psychiatric illness, osteoporosis and cataracts.
Methylprednisolone, prednisolone or prednisone	**Pneumocystis pneumonia prophylaxis:** Consider prophylaxis such as trimethoprim/sulfamethoxazole. **Peptic ulcer prophylaxis:** Consider prophylaxis with histamine-2 receptor antagonist. **Bone health:** Assess baseline fracture risk and consider calcium and vitamin D supplementation.
Targeted release budesonide (Nefecon)	**Capsule intake:** Should be taken whole at least 1 h before breakfast. Capsule should be swallowed whole and not crushed, chewed or opened. **Liver disease:** Avoid in patients with advanced liver disease.

### 5.3 Evidence and utility

STOP-IgA involved patients with an eGFR of ≥30 mL/min/1.73 m^2^ and a urine protein excretion value of ≥0.75 g/day randomized to receive immunosuppression vs. supportive care. Immunosuppression included glucocortocoids for patients with an eGFR ≥ 60 mL/min/1.73 m^2^ or prednisolone with cyclophosphamide/azathioprine for patients with an eGFR of 30–60 mL/min/1.73 m^2^. Although patients receiving immunosuppression had increases of proteinuria remission, there was no significant improvement in long term renal outcomes and adverse events were more frequent in the immunosuppression arm (99).

In the TESTING trial, patients with IgA nephropathy and proteinuria ≥1 g/day and an eGFR of ≥20 mL/min/1.73 m^2^ receiving supportive care were randomized to receive methylprednisolone vs. placebo. The initial methylprednisolone dose of 0.6–0.8 mg/kg/day was reduced to 0.4 mg/kg/day because serious infections were observed. Overall, methylprednisolone significantly reduced proteinuria and was associated with a lower risk of the composite kidney outcome although toxicity remained a key limitation (87).

More recently TRF-budesonide has emerged as an effective therapy for IgA nephropathy. Patients with IgA nephropathy, an eGFR ≥ 35 mL/min/1.73 m^2^ and a urine protein to creatinine ratio > 0.8 g/g (or 24 h urine protein ≥1 g/24 h) were found to have improved kidney outcomes when compared to placebo (100).

When available, TRF-budesonide should be considered in patients with IgA nephropathy with an eGFR ≥ 35 mL/min/1.73 m^2^ and a urine protein to creatinine ratio ≥1.5 g/g especially if they are deemed high risk for progressive loss of kidney function. A 9-month treatment course is a common approach. Systemic glucocorticoids can be considered when targeted release budesonide is not available (45).

## 6 Complement inhibition

### 6.1 Introduction

The role of complement activation in the pathogenesis of IgA nephropathy has been known for decades but until recently, there were no therapeutic options available to target this pathway (101–103). C3 fragments are co-deposited with IgA in almost all biopsy samples of patients with IgA nephropathy and patients with IgA vasculitis with nephritis (104, 105). Proteomic analysis of IgA containing immune complexes in patients with IgA nephropathy indicate the involvement of the alternative and lectin pathways (106). Despite local activation of the alternative complement pathway, typically plasma complement levels are normal in IgA nephropathy, however when they are depressed it tends to be associated with poorer outcomes (107).

Iptacopan, a complement factor B inhibitor, FDA approved for paroxysmal nocturnal hemoglobinuria and C3 glomerulopathy, has received approval for IgA nephropathy. Ravulizumab, a long acting C5 complement inhibitor has also shown promising findings but has not been FDA approved at the time of this paper (108, 109) (Figure 3).

**Figure 3:**
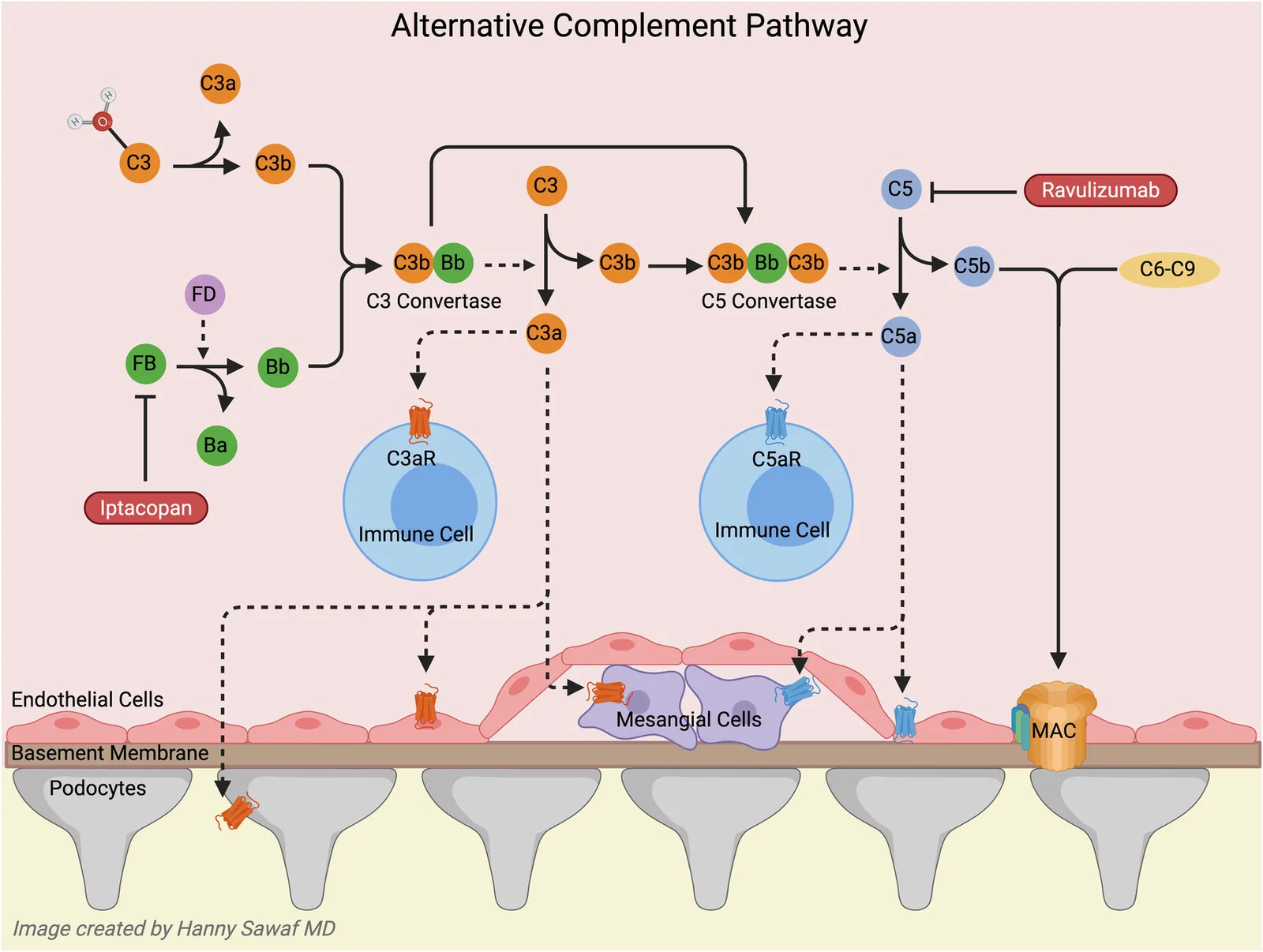
Visual representation on the alternate complement pathway and its role in the progression of glomerular disease.

### 6.2 Practical considerations

The complement system is crucial for fighting encapsulated bacteria such as Streptococcus pneumoniae, Neisseria meningitidis and Haemophilus influenza. Therefore, use of iptacopan requires Neisseria meningitidis vaccination (MenACWY and MenB vaccines or a combined vaccine) prior to the administration of a complement inhibitor. For previously unvaccinated adults consider vaccination against Streptococcus pneumoniae and Haemophilus influenzae type b or a booster for those already vaccinated. Vaccine administration should be given 2 weeks prior to starting therapy. If initiation of complement inhibition is time sensitive, antibiotic prophylaxis (amoxicillin, or ciprofloxacin in PCN allergic patients) is recommended for at least 2 weeks following the first dose of the vaccines. Centers for Disease Control Advisory Committee on Immunization Practices (CDC/ACIP) can be used for further guidance.

Other side effects noted in drug trials include slightly higher rates of upper respiratory infections and lipid disorder however the clinical significance of this is not known (15) (Table 5).

**Table 5:** Practical consideration for iptacopan.

**Medication**	**Practical consideration**
Iptacopan	**Vaccination:** Complete or update vaccination 2 weeks prior to initiation for Neisseria meningitidis subtypes A, C, W, Y and B. Booster vaccination will be required. **Prophylactic Antibiotics:** If treatment cannot be delayed, vaccinate and initiate prophylactic antibiotics for 2 weeks **Unvaccinated Patients**: Consider vaccination against Haemophilus influenza type b and Streptococcus pneumoniae **Lipid:** Periodic lipid monitoring when clinically indicated **REMS:** Required for prescription in the United States

### 6.3 Evidence and utility

The APPLAUSE-IgA trial compared patients with biopsy-confirmed IgA nephropathy with a urine protein excretion value ≥1 g/day despite optimal therapy to receive either iptacopan or placebo. At 9 months the urinary protein excretion was 38% lower with the iptacopan group as compared to placebo (15). At 24 months patients on iptacopan had a favorable eGFR slope at −3.10 mL/min/1.73 m^2^ compared to −6.12 mL/min/1.73 m^2^ with placebo (110).

More recently, ravulizumab, a complement C5 inhibitor has shown promising findings with the I CAN Phase III trial revealing a 46.6% reduction in the 24-hour urine protein at week 34 compared to a 5.6% reduction in patients who received placebo (109). This medication has yet to be FDA approved at the time of this publication.

Iptacopan can be considered in patients with IgA nephropathy and a urine protein to creatinine ratio >1.5 g/g despite maximally tolerated or labeled RAS inhibition. This medication would be considered especially if a patient is at risk of developing adverse effects from steroids and is hesitant about subcutaneous medications. It is not clear whether the intensity of complement staining on biopsy can be used to guide treatment.

## 7 A proliferation-inducing ligand (APRIL) and B-cell activating factor (BAFF) inhibition

### 7.1 Introduction

A proliferation-inducing ligand (APRIL) and B-cell activation factor (BAFF) are related cytokines in the tumor necrosis factor (TNF) superfamily produced by dendritic cells, myeloid cells, and mucosal epithelial cells (111). Signaling related to BAFF and APRIL plays an important role in B-cell survival, maturation, proliferation and class switching (111, 112). BAFF is involved in the survival and development of B cells while APRIL act at later stages relating to function and survival of antigen experienced B cells and plasma cells (113–115).

Sibeprenlimab is a humanized IgG2 monoclonal antibody that selectively binds to and inhibits APRIL. Sibeprenlimab and atacicept are currently the only agents of this class that are currently FDA approved (116). Other agents include telitacicept and povetacicept which are a native human transmembrane activator and calcium modulator and cyclophilin-ligand interactor (TACI)-Fc fusion protein that inhibit both BAFF and APRIL, and zigakibart which is a humanized monoclonal antibody that binds to APRIL (117–120) (Figure 4).

**Figure 4:**
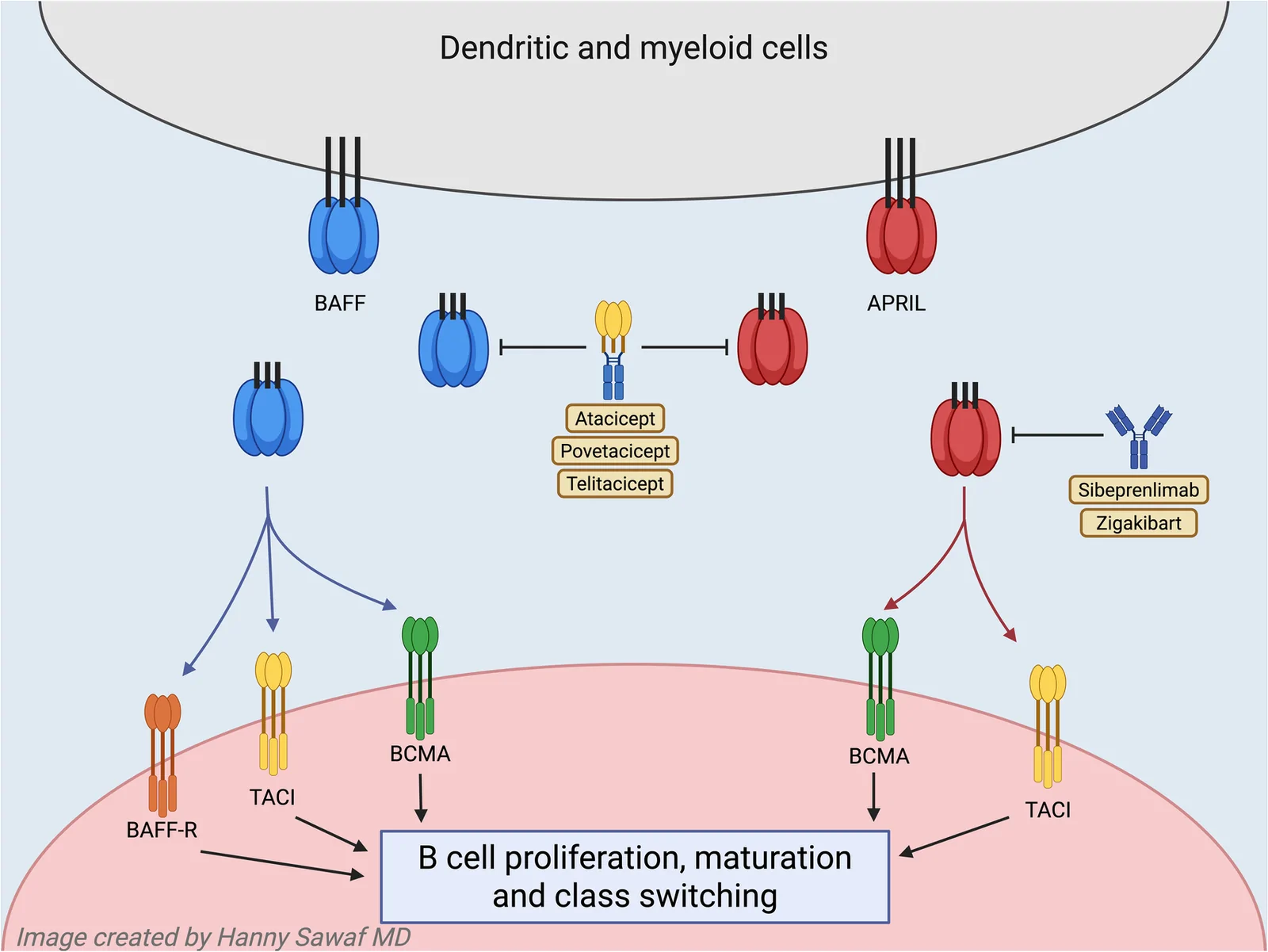
Visual representation of the mechanisms of action of sibeprenlimab, atacicept, telitacicept, povetacicept and zigakibart on a proliferation-inducing ligand (APRIL) and B-cell activation factor (BAFF).

### 7.2 Practical considerations

Given their roles in normal immune function, BAFF and APRIL inhibition results in immunosuppression and has been shown to suppress all antibodies, not just IgA. Therefore, live vaccines are not recommended within 30 days of drug initiation or treatment. Upper respiratory infections have also been described in trials.

These are only available in subcutaneous injections so injection site erythema has also been described with these agents (116, 119). Finally, it may be helpful to check for hypogammaglobulinemia given these agents' effect on antibody-producing cells (121) (Table 6).

**Table 6:** Practical consideration for BAFF/APRIL inhibitors.

**Medication**	**Practical consideration**
BAFF/APRIL inhibition	**Live Vaccination:** Not recommended within 30 days prior to initiation of these therapies or during treatment. **Hypogammaglobulinemia:** Can check levels if clinically indicated **Injection Site Erythema:** Patient education on proper injection technique.

### 7.3 Evidence and utility

The VISIONARY trial compared patients receiving 400 mg of subcutaneous sibeprenlimab every 4 weeks to placebo. Studied patients had biopsy proven IgA nephropathy, an eGFR ≥ 30 mL/min/1.73 m^2^ and a urine protein to creatinine ratio of 0.75 g/d or higher (or a 24-hour urine protein excretion of 1.0 g/day) on maximally tolerated ACE inhibitor or ARB for 3 months. At 9 months the 24 h urinary protein to creatinine ratio had decreased by 50.2% when compared to placebo which had an increase of 2.1% (116).

The ORIGIN trial studied atacicept subcutaneous 150 mg weekly compared to placebo. Patients were required to have an eGFR ≥ 30 mL/min/1.73 m^2^ and a 24-hour urine protein to creatinine ratio of at least 1 g per day despite being on maximum tolerated RAS. At 36 weeks patients receiving atacicept experienced a 45.7% reduction of proteinuria compared to a 6.8% decrease in the placebo group (119). Of the agents mentioned in this section, sibeprenlimab and atacicept are the only one that is FDA approved at the time of this review.

Although sibeprenlimab and atacicept are the only agents currently FDA approved, other medications with similar mechanisms are being investigated. Telitacicept also showed a 58.9% decline of proteinuria at 39 weeks compared to 8.8% with placebo in the TELIGAN trial and even showed evidence when compared to MMF in high-risk IgA nephropathy patients (117, 122). Povetacicept has shown favorable findings in the recently completed RAINER trial (123) while zigakibart also demonstrated clinical safety and efficacy in early phase 1/2 trials (118).

These agents are an important addition to the previously mentioned immunotherapeutic options, providing patients and clinicians further alternatives for the treatment of IgA nephropathy. These medications can be helpful in patients with IgA nephropathy, an eGFR ≥ 30 mL/min/1.73 m^2^ and a urine protein to creatinine ratio of 1 g/d or higher despite maximal RAS inhibition especially in patients who may not tolerate steroid-related side effects or those who would prefer weekly to monthly injections rather than daily oral medications. Choosing which of the BAFF/APRIL agents to prioritize is not entirely known at this time. Although intuitively one could argue to target both BAFF and APRIL vs. only targeting APRIL, there is no evidence to support this.

## 8 Other therapeutic options

Although many of the abovementioned medications for IgA nephropathy have been recently developed, there are other treatment options that could have a role in certain instances.

Mycophenolate mofetil and hydroxychloroquine have both shown potential utility in the Chinese population although there is insufficient evidence to support their utility in patients who are not Chinese or of Chinese background (124–127). There is also evidence that mycophenolate mofetil could be used in addition to BAFF/APRIL inhibition in certain instances (128).

Mycophenolate mofetil, like many other immunosuppressants, is teratogenic so that should be kept in mind when prescribing to women of child bearing age. Other side effects include bone marrow suppression (including neutropenia) for which monitoring blood count with differential may be beneficial. Finally, gastrointestinal effects such as nausea, vomiting, abdominal pain and diarrhea can occur so patients should be educated and aware of these effects. Adverse effects to keep in mind when prescribing hydroxychloroquine include ocular effects which for which ophthalmology monitoring is suggested and QT prolongation for which an electrocardiogram prior to drug initiation could be helpful.

Tonsillectomy has been investigated alone or with steroids to extend kidney survival and increase the likelihoods of partial or complete remission for patients from Japan although its utility in other populations has not been proven (129).

## 9 Future directions

Even with all the therapeutic options added in recent years, there continue to be emerging therapeutic options and avenues. Some of these emerging therapies are those mentioned above that are in early trials as of the time of this publication. That includes the complement inhibitor ravulizumab in addition to BAFF and APRIL targeting therapies such as povetacicept, telitacicept and zigakibart (108, 117, 118).

Potential future options could include chimeric antigen receptor (CAR)-T-cell therapy or bispecific antibodies which have shown some promise in other autoimmune diseases (130, 131). The link between the gut microbiome and IgA nephropathy does raise the possibility of gut microbiome modulation potentially playing a future role (132). Other future developments for the treatment of IgA nephropathy could include biomarker guided precision medicine potentially using Gd-IgA levels, complement deposition patterns and genetic factors to help guide therapy.

Finally, when looking at the current treatment options for IgA nephropathy, future utility including combination and potential synergistic effects of existing agents may prove to be beneficial in the future.

## 10 Conclusion

The recent advances in treatment options for IgA nephropathy provides more options to delay the progression of disease however this also comes with more medications prescribers should be familiar with. Non immunosuppressive therapies such as RAS blockade, SGLT2 inhibitors, and ERA should be utilized to prevent the progression of disease in patients with proteinuric IgA nephropathy. ACE inhibitors and ARBs remain first line treatment and were consistently used in trials looking into other therapeutic options. SGLT2 inhibitors and ERAs can be added for renal protection without a strong focus on immunosuppression. Complement inhibitors, targeted release budesonide and BAFF/APRIL based therapies are all treatment options for patients with IgA nephropathy when immunomodulation is needed to stabilize kidney function. Utilization of these agents in combination with one another is lacking and it is unclear if the risks or benefits of these therapies are additive or attenuated.

Although the availability of these new pharmacologic agents offers clear benefits, it also introduces new challenges in treatment selection. When comparing different immunosuppressive options, it remains unclear how to best use these medications: what order, how to combine, and optimal duration of therapy. These unresolved questions underscore the need for further research and expert led guidelines regarding the different therapeutic approaches for patients with IgA nephropathy (Table 7).

**Table 7:** Summary of medications for IgA nephropathy. eGFR and proteinuria are obtained from trial enrollment data. Many of these trials did not require a “new kidney biopsy” for eligibility.

Medication	eGFR (mL/min)	Urine protein (g/d)	Uses^a^	Comments
ACEi/ARB	Any	Any	All patients that can tolerate	ACEi and ARB should not be combined
SGLT2i	≥20	Any	Add onto ACEi or ARB for proteinuria	Not approved for patients with DM1
ERA/DEARA	≥30	≥1–1.5	Patients with proteinuria who do not require or would not tolerate immunosuppression	Sparsentan should not be combined with ACEi or ARB Can cause volume overload in susceptible patients Liver function should be monitored Can worsen anemia
Glucocorticoids	≥35	≥0.8–1.5	Patients with proteinuria who would benefit from immunosuppression	TRF-budesonide has less systemic effects than systemic glucocorticoids such as prednisone Patients at increased risk of toxicity from systemic glucocorticoids include DM, pre-DM, obesity, latent infections (viral hepatitis and TB), active peptic ulceration, uncontrolled psychiatric illness, osteoporosis, cataracts
Complement inhibitors	≥30–45	≥1–1.5	Patients with proteinuria who would benefit from immunosuppression, especially if at risk for side effects from glucocorticoids	Vaccination for encapsulated organisms required
BAFF/APRIL inhibitors	≥30	≥0.75–1	Patients with proteinuria who would benefit from immunosuppression especially if at risk for side effects from glucocorticoids and for patient preference who may prefer weekly to monthly injections rather than daily oral medications.	Avoid live vaccines 30 days prior to initiation of medication and while on medication
Mycophenolate mofetil (MMF)	≥30^b^	>1	Patients who are Chinese or of Chinese background who would benefit from decreased steroid or steroid sparing immunosuppression	Can cause gastrointestinal effects Complete blood count with differential should be monitored

a Patients with active crescentic disease or rapidly progressive glomerulonephritis may benefit from more aggressive immunosuppressive therapy including cyclophosphamide-based therapies.

b Cutoffs depending on the trial were either an eGFR ≥ 30 or a creatinine <3.4 mg/dL.
